# Supplementary material for: Mid-life social participation in people with intellectual disability: The 1958 British birth cohort study
Source: PLoS One. 2024 May 20;19(5):e0302411. doi: 10.1371/journal.pone.0302411 (PMC11104648; doi:10.1371/journal.pone.0302411)
Supplement: S1 Appendix — (DOCX) [file pone.0302411.s001.docx]

# S1 Appendix. Questionnaires

Table A. Berkman Syme Social Network Index

| **Berkman Syme social network index** | Wording |
| --- | --- |
| Social contact with relatives: | H6. How often do you have regular contact with relatives outside your household, by visits, telephone, letters or emails? In total, is it …  (1)Almost daily; (2) About once a week.  (3) About once a month; (4) Once every few months; (5) Never or almost never;  H7. How often do you visit or are you visited by relatives who live outside your household? In total, is it …  Answer (1)-(5)  H8. How many relatives do you see once a month or more?   1. None; (2) One or two; (3) Three to five; (4) Six to ten; (5) More than ten |
| Social contact with friends | H9. How often do you have regular contact with friends or acquaintances outside your household, by visits, telephone, letters or emails? In total, is it …  Answer (1)-(5)    H10. How often do you visit or are you visited by friends or acquaintances who live outside your household? In total, is it …  Answer (1)-(5)  H11. How many friends or acquaintances do you see once a month or more?  (1)None; (2) One or two; (3) Three to five; (4) Six to ten; (5) More than ten |

Table B. The close person questionnaire at age 44

| **The close person questionnaire at age 44** | **Wording** |
| --- | --- |
| Confiding/emotional support subscale | **How much in the last 12 months…**  H3a.Did this person give you information, suggestions and guidance that you found helpful?  (1)Not at all; (2)A little; (3) Quite a lot; (4)A great deal.  H3c.Did this person make you feel good about yourself?  Answer (1)-(4)  H3e. Did you share interests, hobbies and fun with this person?  Answer (1)-(4)  H4a. Did you want to confide in (talk frankly, share feelings with) this person?  Answer (1)-(4)  H4b. Did you confide in this person?  Answer (1)-(4)  H4c.Did you trust this person with your most personal worries and problems?  Answer (1)-(4)  H5a.Did he/she talk about his/her personal worries with you?  Answer (1)-(4) |

Table C. Control, Autonomy, Self-realization, and Pleasure Scale (CASP-12)

| **CASP-12 Item at age 50** | **Wording** |
| --- | --- |
| 1-3 Control | 1 My age prevents me from doing the things I would like to.  (1) Often; (2) Sometimes; (3) Not often; (4) Never  2 I feel what happens to me is out of my control.  Answer (1)-(4)  3 I feel left out of things  Answers (1)- (4) |
| 4-6 Autonomy | 4 I can do the things I want to do  Answer (1)-(4)  5 Family responsibilities prevent me from doing what want to do  Answer (1)-(4)  6 Shortage of money stops me from doing things I want to do  Answer (1)-(4) |
| 7-9 Self-Realisation | 7 I look forward to each day.  Answer (1)-(4)  8 I feel that my life has meaning.  Answer (1)-(4)  9 On balance, I look back on life with a sense of happiness.  Answer (1)-(4) |
| 10-12 Pleasure | 10 I feel full of energy these days.  Answer (1)-(4)  11 I feel that life is full of opportunities.  Answer (1)-(4)  12 I feel that the future looks good for me.  Answer (1)-(4) |
