# Supplementary material for: Mid-life social participation in people with intellectual disability: The 1958 British birth cohort study
Source: PLoS One. 2024 May 20;19(5):e0302411. doi: 10.1371/journal.pone.0302411 (PMC11104648; doi:10.1371/journal.pone.0302411)
Supplement: S2 Appendix — (DOCX) [file pone.0302411.s002.docx]

# S2 Appendix. Baron and Kenny method and statistical significance tests techniques

We fitted three regression models test paths (a), (b) and (c) as shown in S2 Fig. The possible results are ‘no =partial or full mediation’(Baron & Kenny, 1986; Iacobucci et al., 2007).


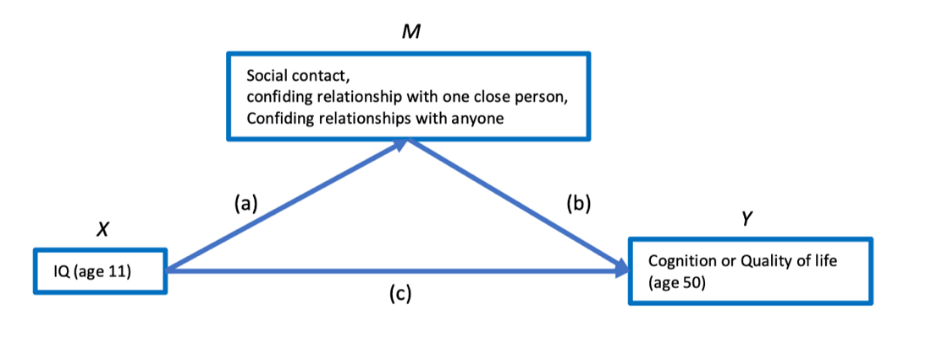


S2 Fig. Progress of mediation analysis

1. $M=\beta_{1}+aX+\varepsilon_{1}$
2. $Y=\beta_{2}+cX+\varepsilon_{2}$
3. $Y=\beta_{3}+c^{'}X+bM+\varepsilon_{3}$

In this study, equation (1) is to test IQ at age 11 significantly predicted social participation and significantly predicted cognition or quality of life at age 50. As the relationships were shown to be significant, we then fitted the data into equation (2), with the outcomes being cognition or quality of life at age 50 (as shown in S2 Fig). After that, we fitted the data into equation (3) to see if the effect of IQ in childhood on cognition or quality of life at age 50 is mediated by social participation. We repeated this process for different forms of social participation (social contact frequency at 44, confiding relationships with one close person at 44, and confiding relationships with any person at 50). We analysed different groups (people with intellectual disability, and people with IQ above 85) separately.

After that, we did Sobel’s z test and bootstrapping to test the statistical significance of the indirect effect of mediators. Baron and Kenny’s approach is commonly followed by Sobel’s (1987) z-test^2^ to test the significance of (c-c’).

$$z=\frac{a\times b}{\sqrt{b^{2}s_{a}^{2}+a^{2}s_{b}^{2}}}$$

To gauge the effect size of an indirect effect, we calculated the ratio of the indirect effect to the total effect (RIT) using the formula below:

$$RIT=\frac{Indirect Effect}{Indirect Effect+Direct Effect}=\frac{a\times b}{\left( a\times b \right)+c}$$

Additionally, Zhao et al.(Zhao et al., 2010) suggested that Sobel’s z-test assumed the sampling distribution is symmetrical by using a normal approximation, but the distribution of can be skewed. Therefore, we used bootstrapping technique to further test the significance of the indirect effect by computing and collecting the indirect effects from each of the n samples (in this study, n=200).

In this study, according to S2 Fig, equation (1) is to test IQ at age 11 significantly predicted social participation and significantly predicted cognition or QoL at age 50. As the relationships were significant, we then fitted the data into equation (2), with the outcomes being cognition or QoL at age 50 (as shown in S2 Fig). After that, we fitted the data into equation (3) to see if the effect of IQ in childhood on cognition or QoL at age 50 is mediated by social participation. We repeated this process for different forms of social participation (social contact frequency at 44, confiding relationships with one close person at 44, and confiding relationships with any person at 50). We analysed different groups (people with ID, and people with IQ above 85) separately. The mediation effect of social participation on the relationships between IQ in childhood and cognition or QoL at age 50 is summarised in Table 3.

In Table 3, the direct effect is the effect of IQ in childhood on the cognition or QoL at age 50 absent social participation. The indirect pathway is the effect of IQ in childhood on cognition or QoL at age 50 through social participation.
