# Supplementary material for: Mid-life social participation in people with intellectual disability: The 1958 British birth cohort study
Source: PLoS One. 2024 May 20;19(5):e0302411. doi: 10.1371/journal.pone.0302411 (PMC11104648; doi:10.1371/journal.pone.0302411)
Supplement: S1 Table — (DOCX) [file pone.0302411.s003.docx]

# S3 Table. The mediation effect of social participation on the relationship between IQ at age 11 and cognition at age 50.

|  | People with IQ above 85 | | | | | People with borderline intellectual functioning | | | | | People with mild intellectual disability | | | | |
| --- | --- | --- | --- | --- | --- | --- | --- | --- | --- | --- | --- | --- | --- | --- | --- |
|  | (1)  Social network index | (2) Relatives’ subscale | (3) Friends’ subscale | (4)  confiding/emotional support from the closest person | (5)  Confiding relationships with anyone | (1)  Social network index | (2)  Relatives’ subscale | (3)  Friends’ subscale | (4)  confiding/emotional support from the closest person | (5)  Confiding relationships with anyone | (1)  Social network index | (2)  Relatives’ subscale | (3)  Friends’ subscale | (4)  confiding/emotional support from the closest person | (5)  Confiding relationships with anyone |
| N | 4322 | 4715 | 5104 | 5537 | 7071 | 548 | 601 | 610 | 653 | 973 | 23 | 29 | 26 | 30 | 57 |
| *Paths:* |  |  |  |  |  |  |  |  |  |  |  |  |  |  |  |
| *(c) X → Y*  *(P value)*   1. *(a) X →M*   *(P value)*   1. *(b) M → Y*   *(P value)* | 0.03 | 0.03 | 0.03 | 0.03 | 0.03 | 0.03 | 0.03 | 0.03 | 0.04 | 0.03 | 0.06 | 0.08 | 0.07 | 0.09 | 0.08 |
|  | (<0.001) | (<0.001) | (<0.001) | (<0.001) | (<0.001) | (0.002) | (0.001) | (<0.001) | (<0.001) | (<0.001) | (0.437) | (0.106) | (0.336) | (0.076) | (0.034) |
|  | -0.01 | -0.02 | 0.01 | 0.003 | 0.002 | -0.04 | -0.01 | -0.03 | 0.03 | 0.03 | -0.48 | -0.02 | -0.29 | -0.24 | 0.06 |
|  | (0.220) | (<0.001) | (<0.001) | (0.541) | (0.001) | (0.299) | (0.551) | (0.284) | (0.484) | (0.001) | (0.050) | (0.837) | (0.066) | (0.387) | (0.054) |
|  | -0.01 | -0.02 | 0.01 | 0.01 | 0.07 | 0.01 | 0.01 | 0.02 | 0.03 | 0.09 | 0.11 | 0.10 | 0.22 | 0.01 | 0.19 |
|  | (0.232) | (0.004) | (0.248) | (0.002) | (<0.001) | (0.184) | (0.413) | (0.207) | (<0.001) | (0.012) | (0.103) | (0.316) | (0.016) | (0.707) | (0.261) |
| Indirect effect |  | <0.001 |  |  | <0.001 |  |  |  |  | 0.002 |  |  |  |  |  |
| (P value) |  | (<0.001) |  |  | (0.009) |  |  |  |  | (0.042) |  |  |  |  |  |
| Sobel’s z value |  | 2.71 |  |  | 2.62 |  |  |  |  | 2.029 |  |  |  |  |  |
| Bootstrapping [95% C.I.] |  | [<0.001,0.001] |  |  | [<0.001,0.003] |  |  |  |  | [<0.001,0.005] |  |  |  |  |  |
| Direct effect |  | 0.03 |  |  | 0.03 |  |  |  |  | 0.03 |  |  |  |  |  |
| (P value) |  | (p<0.001) |  |  | (<0.001) |  |  |  |  | (<0.001) |  |  |  |  |  |
| RIT(%) | No mediation | 1.2, partial mediation | No mediation | No mediation | 0.5, partial mediation | No mediation | No mediation | No mediation | No mediation | 6.4, partial mediation | No mediation | No mediation | No mediation | No mediation | No mediation |

Notes: ^a^ N = number of observations. ^b^ C.I.=Confidence interval. ^c^ As described above. If |$z|$ above 1.96, then the mediation $(c-c^{'})$ or $a\times b$ is statistically significant at 0.05. ^d^ *X* = participants’ IQ test scores at age 11, *M* = social participation, including social contact with relatives/friends, confiding/emotional support from the closest person, and confiding relationships with anyone, *Y* = participant’s cognition at age 50.
