# Supplementary material for: Mid-life social participation in people with intellectual disability: The 1958 British birth cohort study
Source: PLoS One. 2024 May 20;19(5):e0302411. doi: 10.1371/journal.pone.0302411 (PMC11104648; doi:10.1371/journal.pone.0302411)
Supplement: S2 Table — (DOCX) [file pone.0302411.s004.docx]

# S4 Table. The mediation effect of social participation on the relationship between IQ at age 11 and QoL at age 50.

|  | People with IQ above 85 | | | | | People with borderline intellectual functioning | | | | | People with mild intellectual disability | | | | |
| --- | --- | --- | --- | --- | --- | --- | --- | --- | --- | --- | --- | --- | --- | --- | --- |
|  | (1) Social network index | (2) Relatives’ subscale | (3) Friends’ subscale | (4)  confiding/emotional support from the closest person | (5)  Confiding relationships with anyone | (1)  Social network index | (2)  Relatives’ subscale | (3)  Friends’ subscale | (4)  confiding/emotional support from the closest person | (5)  Confiding relationships with anyone | (1)  Social network index | (2)  Relatives’ subscale | (3)  Friends’ subscale | (4)  confiding/emotional support from the closest person | (5)  Confiding relationships with anyone |
| N | 4076 | 4450 | 4825 | 5243 | 6550 | 502 | 548 | 555 | 593 | 850 | 17 | 22 | 19 | 23 | 46 |
| *Paths:* |  |  |  |  |  |  |  |  |  |  |  |  |  |  |  |
| *(c) X → Y*  *(P value)*   1. *X →M*   *(P value)*   1. *M → Y*   *(P value)* | 0.03 | 0.03 | 0.03 | 0.03 | 0.03 | 0.08 | 0.10 | 0.13 | 0.15 | 0.16 | 0.42 | -0.33 | 0.57 | -0.37 | -0.15 |
|  | (<0.001) | (<0.001) | (<0.001) | (<0.001) | (<0.001) | (0.243) | (0.154) | (0.062) | (0.030) | (0.006) | (0.501) | (0.366) | (0.328) | (0.286) | (0.357) |
|  | -0.003 | -0.02 | 0.01 | 0.004 | 0.002 | -0.02 | -0.01 | -0.01 | 0.04 | 0.03 | -0.69 | -0.06 | -0.38 | -0.30 | 0.05 |
|  | (0.460) | (<0.001) | (<0.001) | (0.365) | (0.005) | (0.666) | (0.787) | (0.554) | (0.418) | (0.001) | (0.017) | (0.539) | (0.055) | (0.287) | (0.038) |
|  | 0.25 | 0.24 | 0.33 | 0.27 | 2.14 | 0.20 | 0.15 | 0.43 | 0.21 | 1.68 | 0.73 | 0.61 | 0.80 | 0.21 | 1.18 |
|  | (<0.001) | (<0.001) | (<0.001) | (<0.001) | (<0.001) | (0.008) | (0.235) | (<0.001) | (<0.001) | (<0.001) | (0.242) | (0.511) | (0.301) | (0.457) | (0.275) |
| Indirect effect |  | -0.004 | 0.004 |  | 0.004 |  |  |  |  | 0.043 |  |  |  |  |  |
| (P value) |  | (<0.001) | (<0.001) |  | (0.005) |  |  |  |  | (0.003) |  |  |  |  |  |
| Sobel’s z value |  | -4.18 | 4.66 |  | 2.79 |  |  |  |  | 2.97 |  |  |  |  |  |
| Bootstrapping [95% C.I.] |  | [-0.006, -0.002] | [0.002,0.006] |  | [0.001,0.007] |  |  |  |  | [0.014,0.071] |  |  |  |  |  |
| Direct effect |  | 0.04 | 0.02 |  | 0.03 |  |  |  |  | 0.11 |  |  |  |  |  |
| (P value) |  | (<0.001) | (<0.001) |  | (<0.001) |  |  |  |  | (0.041) |  |  |  |  |  |
| RIT (%) | No mediation | -12.5, partial mediation | 14.4, partial  mediation | No mediation | 13.4, partial mediation | No mediation | No mediation | No mediation | No mediation | 27.4, partial mediation | No mediation | No mediation | No mediation | No mediation | No mediation |

Notes: ^a^ N= number of observations. ^b^ C.I.=Confidence interval. ^c^As described above. If |$z|$ above 1.96, then the mediation $(c-c^{'})$ or $a\times b$ is statistically significant at 0.05. *X* = participants’ IQ test scores at age 11, *M* = social participation, including social contact with relatives/friends, confiding/emotional support from the closest person, and confiding relationships with anyone, *Y* = participant’s QoL at age 50.
