# Supplementary material for: Mid-life social participation in people with intellectual disability: The 1958 British birth cohort study
Source: PLoS One. 2024 May 20;19(5):e0302411. doi: 10.1371/journal.pone.0302411 (PMC11104648; doi:10.1371/journal.pone.0302411)
Supplement: S3 Table — (DOCX) [file pone.0302411.s005.docx]

# S5 Table. IQ test scores, cognitive test scores and social participation in study participants

| Variable name | All Participants | | People with IQ above 85 | | People with borderline intellectual functioning | | People with mild intellectual disability | |  | |
| --- | --- | --- | --- | --- | --- | --- | --- | --- | --- | --- |
|  | N | %, Mean (SD) | N | %, Mean (SD) | N | %, Mean (SD) | N | %, Mean (SD) | |  |
| **IQ tests Subscores at age 11** |  |  |  |  |  |  |  |  | |  |
| *Reading* | 14094 | 16.0(6.3) | 11625 | 17.7(5.2) | 2234 | 8.2(3.1) | 235 | 2.2(2.9) | |  |
| *Maths* | 14094 | 16.6(10.4) | 11625 | 19.3(9.3) | 2234 | 4.3(2.9) | 235 | 0.6(1.5) | |  |
| *Copying* | 14094 | 8.3(1.5) | 11625 | 8.6(1.3) | 2234 | 7.4(1.3) | 235 | 4.5(2.5) | |  |
| *General ability* | 14094 | 42.9(16.2) | 11625 | 47.8(13.0) | 2234 | 21.4(6.8) | 235 | 8.8(7.9) | |  |
| *Synthesised IQ score ^c^* | 14094 | 100(15) | 11625 | 104.7(11.8) | 2234 | 78.5(3.8) | 235 | 63.5(6.0) | |  |
| **Cognition at age 50** |  |  |  |  |  |  |  |  | |  |
| *letter cancellation task* | 8178 | 47.7 (12.8) | 7136 | 48.1(12.8) | 985 | 45.0(11.8) | 57 | 43.5(14.0) | |  |
| *animal naming* | 8351 | 22.4(6.3) | 7268 | 22.9(6.2) | 1018 | 18.9(5.7) | 65 | 16.6(5.3) | |  |
| *word recall immediately* | 8351 | 6.6(1.5) | 7268 | 6.7(1.4) | 1018 | 5.7(1.4) | 65 | 4.8(2.0) | |  |
| *word* *recall delayed* | 8301 | 5.4(1.8) | 7224 | 5.6(1.8) | 1012 | 4.3(1.8) | 65 | 3.4(2.1) | |  |
| ***Berkman-Syme social network index (age 44)*** | 5651 | 22.6(3.1) | 4934 | 22.6(3.1) | 682 | 22.6(3.4) | 35 | 23.5(3.1) | |  |
| *Relatives contact* | 6182 | 11.1(1.9) | 5391 | 11.1(1.9) | 748 | 11.3(2.1) | 43 | 11.8(1.9) | |  |
| *Friends contact* | 6650 | 11.5(2.2) | 5848 | 11.5(2.2) | 761 | 11.2(2.2) | 41 | 11.8(2.1) | |  |
| ***The closest person*** |  |  |  |  |  |  |  |  | |  |
| *-Husband/wife/partner* | 5130 | 75.5 | 4647 | 76.1 | 466 | 71.8 | 17 | 47.2 | |  |
| *-boyfriend/girlfriend* | 250 | 3.7 | 224 | 3.7 | 25 | 3.9 | 1 | 2.8 | | |
| *-parents* | 278 | 4.1 | 232 | 3.8 | 36 | 5.6 | 10 | 27.8 | |  |
| *-Siblings* | 239 | 3.5 | 204 | 3.3 | 33 | 5.1 | 2 | 5.6 | | |
| *-Children* | 241 | 3.6 | 210 | 3.4 | 31 | 4.8 | 0 | 0 | | |
| *-Other relatives* | 33 | 0.5 | 25 | 0.4 | 8 | 1.2 | 0 | 0 | | |
| *-Neighbour* | 20 | 0.3 | 16 | 0.3 | 3 | 0.5 | 1 | 2.8 | |  |
| *-Friend from work* | 476 | 7.0 | 89 | 1.5 | 7 | 1.1 | 0 | 0 | |  |
| *-Other friends* | 96 | 1.4 | 436 | 7.1 | 38 | 5.9 | 2 | 5.6 | |  |
| *-Others* | 29 | 0.4 | 24 | 0.4 | 2 | 0.3 | 3 | 8.3 | |  |
| ***Confiding/emotional support from the closest person at age 44*** | 7202 | 15.2(4.1) | 6350 | 15.3(4.0) | 807 | 14.7(4.4) | 45 | 15.0(5.1) | |  |
| ***Confiding relationships with anyone (age 50)*** | 8385 | 2.6 (0.0.7) | 7287 | 2.7(0.7) | 1026 | 2.5(0.9) | 72 | 2.5(0.9) | |  |
| **QoL at age 50** | 7482 | 26.1(5.8) | 6579 | 26.3(5.7) | 857 | 25.0(6.1) | 46 | 23.0(5.9) | |  |

Notes: ^a^ N= number of observations.
